# Prenatal counseling for heart disease: Perception of understanding and communication gaps

**DOI:** 10.1038/s41372-025-02365-3

**Published:** 2025-08-21

**Authors:** Katherine J. DeWeert, Melissa K. Cousino, Sunkyung Yu, Sarah Gelehrter

**Affiliations:** Department of Pediatrics, University of Michigan, Ann Arbor, MI USA

**Keywords:** Paediatrics, Patient education, Outcomes research

## Abstract

**Background:**

Research on parental understanding following prenatal counseling for congenital heart disease (CHD) is limited. We aimed to evaluate parental knowledge of their fetus’ CHD diagnosis and congruence between physician’s and parent’s assessments of parental understanding.

**Methods:**

Paired surveys of pediatric cardiologists and parents of fetuses requiring neonatal heart surgery assessed parental knowledge and perceived understanding of diagnosis, treatment and complications. Gwet’s agreement coefficients (GAC) were used to examine congruence.

**Results:**

There was good congruence between 40 participating parents and cardiologists regarding knowledge of the diagnosis (GAC 0.89), need for neonatal surgery (GAC 0.81), need for further surgeries and lifelong care (GAC 0.66 and 0.65). While cardiologists perceived parents understood risk of mortality and neurodevelopmental outcomes, parental knowledge was poor (GAC −0.04 and 0.05, respectively).

**Conclusion:**

For many aspects of counseling, parental knowledge is congruent with cardiologist’s perceptions; however, communication gaps exist for knowledge of mortality and other longer-term outcomes.

## Introduction

Advancements in routine obstetric screening have led to an increase in the prenatal diagnosis of congenital conditions, providing more opportunities for prenatal counseling [1–4]. Despite progress in clinical medicine, congenital heart disease (CHD) remains the leading cause of infant mortality related to birth defects and is associated with significant morbidity among survivors. With an incidence of approximately 6 per 1,000 live births, CHD is the most common congenital anomaly [1–4]. However, there is limited evidence on effective prenatal counseling techniques for CHD, and studies have identified significant variations in counseling practices among pediatric cardiologists [2, 4–8].

A retrospective study surveying parents of older children with CHD reported that parents preferred more information than cardiologists provided [6, 9]. However, parental knowledge needs are dynamic and likely to change as their child grows. It is likely that parental information needs during the initial prenatal counseling session differ from those years following a diagnosis. Goals of prenatal counseling include: providing an accurate diagnosis of the malformation, providing a clear picture of the prognosis, outlining management and treatment options that are available, and helping parents reach decisions concerning the form of management that is best for them during often-stressful initial prenatal counseling visits [1, 4].

Recent studies on parental perspectives of prenatal counseling emphasized the variability by which families interpret counseling terminology and physician demeanor, which in turn affects parental understanding of their child’s heart disease and consequently their decisions regarding treatment [6, 7, 10]. The lack of parental understanding in some circumstances could be counteracted by accurate assessment of parental acquisition of knowledge by the fetal cardiologist and improved communication follow-up To better inform prenatal CHD counseling practices, the primary aims of this study were (1) to determine parental understanding and knowledge retention of key counseling topics and presence of communication gaps between the family and the physician, and (2) to assess parental satisfaction after the initial prenatal counseling visit for complex CHD requiring neonatal surgery. Secondary aims included evaluating whether parents had insight into their own knowledge gaps and determining the accuracy of cardiologist perceptions of parental understanding.

## Methods

This institutional review board approved study involving paired surveys of the pediatric cardiologist and parents of fetuses with diagnosis of complex CHD. This study was conducted at a single, Midwest, high-volume pediatric cardiac center from June 2020 to June 2021. Demographics on all parents approached were collected and included maternal age, gestational age, insurance, maternal education level, race and ethnicity, cardiac diagnosis and other major noncardiac or genetic diagnoses. Parents that met criteria for the study were greater than 18 years of age, had a baby with a fetal echocardiogram-confirmed diagnosis of complex CHD that would require neonatal surgery, and underwent their first fetal cardiology counseling session at the study site.

### Survey development

Complementary parent-completed and cardiologist-completed surveys were developed by the study team. Topics on the survey related to parental knowledge acquisition were developed following literature review, and in accord with a prenatal counseling checklist developed in collaboration with a National Pediatric Cardiology Quality Improvement Collaborative committee that includes pediatric cardiologists, advanced practice nurses, and families [1, 2, 4, 6, 8, 11]. In addition, survey refinement and finalization were completed via expert consensus with a team involving fetal cardiology specialists, and a child psychologist that specializes in the psychosocial care of fetal and pediatric cardiology patients and their families. The following topics were included in the surveys: fetal cardiac diagnosis, location of delivery, requirement of intensive care unit (ICU) admission after delivery, surgical management, number of lifetime surgeries and whether the surgical repair were palliative or curative, requirement of long-term cardiac care, mortality and morbidity associated with the diagnosis, risk of decreased exercise tolerance, and risk of neurodevelopmental delays.

### Parental survey

The parental questionnaire took approximately 10–15 min to complete and included 25–30 multiple choice questions, and one free-text question regarding fetal diagnosis. The questions were designed to evaluate both parental perception of knowledge as well as objective understanding by directly comparing it to the cardiologist survey (see supplementary information). The 8-item evidence-based CSQ-8 (Client Satisfaction Questionnaire) was used to evaluate parental satisfaction of their initial prenatal counseling visit [12]. Eligible parent participants were approached via phone one to two weeks after their initial visit. Informed consent was obtained, and participating parents could opt to complete study surveys electronically via email or on an iPad provided at the follow-up visit four to six weeks after their initial visit.

### Cardiologist surveys

The cardiologist-completed survey was developed to complement the parental survey to determine perception of parental acquisition of knowledge of various topics after the initial visit. Our institution’s standard practice is to ensure that a nurse coordinator and a pediatric cardiologist are present at every fetal counseling visit. The nurse coordinator records a note indicating what topics the cardiologist addressed at the visit. This serves as a reference for the pediatric cardiologist to verify the topics discussed at each session. The survey was completed after the initial consultation and included 18 multiple choice questions, taking approximately 5 min to complete.

### Statistical analysis

Standard descriptive statistics were reported using frequency with percentage (%) for categorical variables and mean ± standard deviation or median with interquartile range (IQR) for continuous variable, depending on distributional assumptions. Demographics on mothers who completed the survey vs. those did not were compared using Chi-square test or Fisher’s exact test for categorical variables and Wilcoxon rank sum test for continuous variables. Congruences between cardiologist and parental survey responses on perceptions of parental understanding and knowledge acquisition and communication gaps were assessed using Gwet’s (AC1) agreement coefficient (GAC); GAC < 0.2 indicating poor agreement; 0.2–0.4 indicating fair agreement, 0.4–0.6 indicating moderate agreement; 0.6–0.8 indicating good agreement and > 0.8 indicating excellent agreement [13, 14]. Additionally, univariate association between duration of counseling and parent satisfaction was examined using Wilcoxon rank sum test. All analyses were performed using SAS version 9.4 (SAS Institute, Cary, NC, USA), with a statistical significance level of 0.05 using two-sided tests.

## Results

### Demographics

Of 79 parents that were approached for the study, 40 (51%) completed the survey. About 90% of parents approached for the study had someone accompany them to the counseling visit and 85% brought their partner as their support. All patients in the study described English as their first language. Mothers that completed the survey were older than those who did not (median maternal age 32.1 vs. 28.1 years, *p* = 0.02), presented at a younger gestational age (median 24.9 weeks vs. 27.0 weeks, *p* = 0.03), and were more likely to have private insurance (85% vs. 54%, *p* = 0.001). There were no other significant differences in demographics between participants and non-participants. Nine cardiologists participated in the counseling study with an average of 11.8 years of experience (range 1–26 years). The median counseling time was 40 min (IQR 35–50 min).

Maternal demographics and fetal data for those who completed the survey are shown in Table 1. A majority (78%) of survey respondents had at least a college degree, with 28% having an advanced degree. The most common prenatal diagnoses were aortic arch obstruction (28%), transposition of the great arteries (23%), and single ventricle lesion requiring staged surgical palliation (23%). Four fetuses (10%) had major non-cardiac anomalies and 5 fetuses (13%) had a prenatal genetic diagnosis confirmed by amniocentesis which included DiGeorge Syndrome and Trisomy 21.

**Table 1:** Maternal and fetal demographics (*N* = 40).

Characteristic	
Gestational age at visit, weeks	24.9 (21.9–28.8)*
Maternal age at visit, years	32.1 (27.5–35.8)*
Maternal Race	
White/Caucasian	29 (73%)
Black/African American	4 (10%)
Asian	4 (10%)
Multiracial	2 (5%)
Other	1 (3%)
Maternal Ethnicity: Hispanic	4 (10%)
Insurance: Private	34 (85%)
Highest Level of Maternal Education	
High School	8 (20%)
Technical School	1 (3%)
College	20 (50%)
Graduate School	11 (28%)
Fetal Cardiac Diagnosis	
Aortic Arch Obstruction	11 (28%)
Borderline Left Heart	4 (10%)
Tetralogy of Fallot	5 (13%)
Single Ventricle Heart Disease	9 (23%)
Transposition of the Great Arteries	9 (23%)
Pulmonary Atresia	2 (5%)
Fetal Genetic Anomaly	5 (13%)
Fetal Non-cardiac abnormality	4 (10%)

^*^Data are presented as *N* (%) for categorical variables and median (interquartile range) for continuous variables.

### Perception of parental understanding

**Fig. 1:**
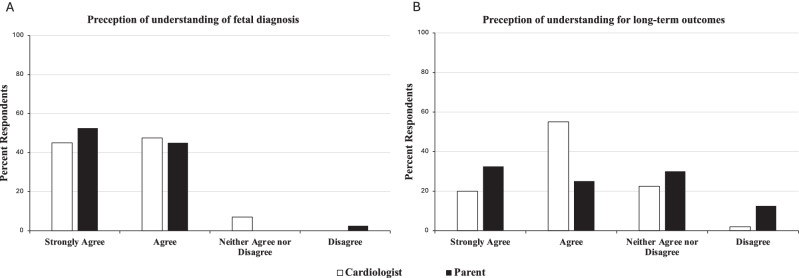
Self and cardiologist perception of parental understanding of key counseling topics. Agreement of parent and cardiologist with statement that parent had “good understanding” of fetal diagnosis **A** and long-term outcomes **B** following initial consultation. Congruence of self-perception and cardiologist perception of parental understanding was excellent (GAC 0.9) for fetal diagnosis, and fair (GAC 0.31) for long-term outcomes.

We defined perception of parental understanding as the subjective assessment of parental understanding by both cardiologist-and parental reported surveys. There was very good congruence (GAC = 0.9) between the self-perception and cardiologists’ perception of parental understanding of the cardiac diagnosis (Fig. 1, A): 97% of parents either strongly agreed or agreed that they understood the fetal diagnosis after their initial counseling session, while 92% of cardiologists either strongly agreed or agreed that the parent understood their fetal diagnosis after their initial counseling session. Only one parent stated they disagreed that they understood the fetal diagnosis after the initial counseling session and this response corresponded to a fetal diagnosis of borderline left-sided structures, a diagnosis of ambiguity. There was less congruence (GAC = 0.31) of perception of parental understanding of long-term outcomes: 75% of cardiologists and 57% of parents either agreed or strongly agreed that parents had a good understanding of long-term outcomes (Fig. 1, B).

### Parental knowledge acquisition and communication gaps

**Fig. 2:**
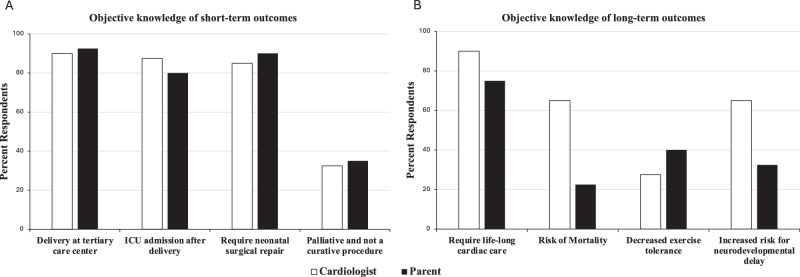
Parental knowledge acquisition. Histogram showing percent of respondents who reported that a topic was discussed during initial consultation. Short-term outcomes are shown in panel **A** and long-term outcomes in **B**.

Knowledge acquisition was an objective measure of congruence defined as information provided by the cardiologist and subsequently described by the parent. All parents were able to accurately describe their child’s fetal diagnosis using a free text response. Topics related to delivery and immediate neonatal care —requiring delivery at a tertiary care center, need for cardiac ICU admission and need for neonatal surgery—had excellent knowledge acquisition congruence (GAC > 0.8) between parents and cardiologists (Fig. 2, A).

There was less knowledge acquisition congruence between parents and cardiologists on topics related to long-term outcomes (Fig. 2, B). The topic of surgery being a complete repair vs. staged palliation was discussed in all cases with moderate agreement (GAC = 0.63) between the parents and the cardiologists. In cases where complete surgical repair was discussed, 67% (18/27) of parents demonstrated understanding. In cases where surgical palliation was discussed, 77% (10/13) demonstrated understanding, while 15% thought that surgery was curative. Ninety-percent of cardiologists (36/40) responded that the child would require lifelong cardiac care despite surgical repair, which had moderate congruence (GAC = 0.65) with the parental understanding of their child’s need for lifelong cardiac care.

Approximately two-thirds (65%; 26/40) of cardiologists discussed the risk of neonatal mortality associated with the fetal diagnosis during the initial counseling visit. Cardiologists were signifcantly more likely to discuss neonatal mortality if they estimated it to be >5%, in which case it was discussed in 95% (18/19) of consultations compared to 38% (8/21) of consultations when neonatal mortality was estimated at < 5% (*p* = 0.0002). Parental understanding of mortality risk was low (GAC = −0.04).

The cardiologist addressed the impact of CHD on neurodevelopmental outcomes, such as learning disabilities and developmental delays, in 65% (26 out of 40) of cases. However, 19% (5 out of 26) of those parents did not recall this discussion. In the 14 cases where the impact of CHD on neurodevelopmental outcomes was not discussed per cardiologist report, 43% (6/14) of parents responded that the cardiologist had discussed the issue, and specifically, recalled that neurodevelopmental delays were unlikely. The impact of CHD on exercise tolerance was discussed by the cardiologist in 28% (11/40) of consultations; however, 40% (16/40) of parents reported that it was discussed (GAC = 0.34).

**Fig. 3:**
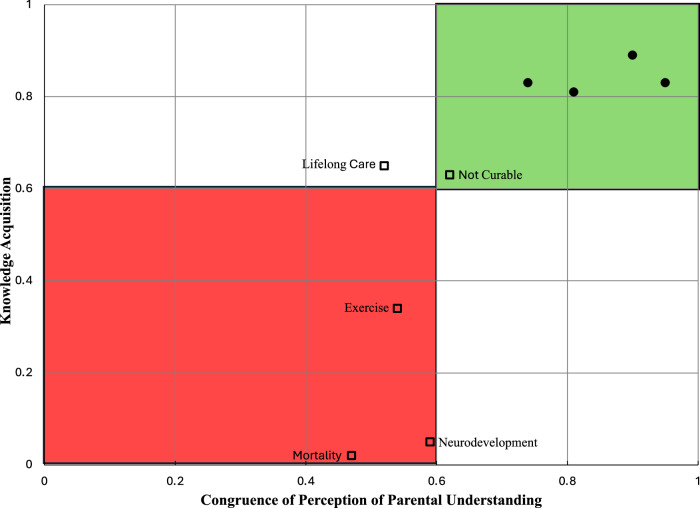
Communication Gaps. Congruence of perception of parental understanding by the cardiologist and parent (x-axis) to demonstrated parental knowledge acquisition of topics covered during the consultation (y-axis) using a GAC of 0.6 (good agreement) as a threshold for adequate congruence. Topics related to short-term outcomes are shown in closed circles; topics related to long-term outcomes are shown in open squares. Data points in the top-right quadrant (shown in green) show no communication gap, while those in the bottom-left quadrant (shown in red) show a communication gap with both parents and cardiologist perceiving the parents understood the information, but a lack of demonstrated parental knowledge acquisition. In the other 2 quadrants (shown in white), there is awareness of the potential communication gap as either parent or cardiologist perceives a lack of parental understanding.

A communication gap occurs when there is a gap between the shared perception of understanding and the acquisition of knowledge. Figure 3 illustrates the alignment between cardiologist and parental perceptions of parental understanding and knowledge acquisition during consultations, using a GAC of 0.6 as the threshold for good agreement. The green top-right quadrant indicates no communication gap, with strong congruence and knowledge acquisition. The red bottom-left quadrant signifies a communication gap, where both parties perceive parental understanding as lacking, resulting in poor knowledge acquisition. Key communication gaps were noted in topics such as diagnosis-related mortality, exercise tolerance, and neurodevelopmental outcomes. The other two quadrants (white) highlight awareness of potential communication gaps, with one party perceiving a lack of understanding. This reveals a perceived lack of understanding regarding the cardiac diagnosis that necessitates lifelong care.

After the initial counseling visit, 70% (28/40) of parents reported that they looked for additional resources for information regarding their child’s diagnosis. Among those 28 families, 86% reviewed other medical websites, 54% used support groups for family with children with the same diagnosis, and 61% of families used social media such as Facebook or Instagram.

### Parental satisfaction with consultation

All parents endorsed satisfaction with their initial counseling session and stated they would return to the program if they needed counseling again in the future. Thirty-two parents (80%) described the initial counseling session as excellent and felt that the counseling session met all of their needs. Overall, cardiologists had a good perception of parental satisfaction with fetal counseling session (GAC = 0.92). There was no association between duration of counseling and parental satisfaction or perception of understanding (*p* = 0.21).

## Discussion

Communication and understanding are critical in terms of fetal CHD care. Initial fetal counseling sessions often set the stage for treatment decision making and the heart disease journey. To our knowledge, this is the first study evaluating parental perception of understanding and demonstrated knowledge acquisition after prenatal counseling in complex CHD. Findings highlighted that parents often have good understanding of fetal diagnosis and immediate neonatal care needs; however, gaps exist with regards to parental understanding and cardiologist communication specific to longer-term outcomes.

In this study, most parents perceived themselves as having a good understanding of their child’s prenatal diagnosis (97%), which correlated with the cardiologists’ interpretation. Our study demonstrated that parental objective knowledge corresponded with their perceived understanding on the fetal cardiac diagnosis, as well as other topics related to delivery and immediate neonatal care such as delivery location, requirement of ICU admission, and the need for neonatal surgery. Cardiologists accurately perceived these high levels of parental understanding.

While strong congruence between parents and cardiologists was observed regarding these immediate neonatal needs, various gaps in communication and understanding were found specific to longer-term outcomes. When evaluating long-term outcomes associated with the fetal diagnosis, only 60% of parents perceived themselves as having a good understanding and 12.5% felt they had a poor understanding. Moreover, findings highlight that parents may have gaps in their perceptions and understanding. For example, in 35% of cases, the cardiologist reported having not discussed neurodevelopmental outcomes with the parents, yet about half of those parents reported the topic had been discussed. Similarly, only 28% of cardiologists reported discussion of the association between the cardiac diagnosis and future exercise intolerance, yet 40% of parents endorsed being counseled on exercise intolerance. This suggests that when there is a gap of information in counseling, parents potentially assume their own answers.

There are likely various contributors to the communication and understanding gaps demonstrated in the current study. Previous studies have demonstrated a complex relationship between stress and memory. Highly stressful situations can result in decreased memory performance. Forty to 80% of medical information provided by healthcare practitioners is forgotten immediately and almost half of that information is remembered incorrectly [15–17]. A previous retrospective study showed that although prenatal counseling was associated with marginally increased parental understanding at neonatal ICU discharge, only ~50% of parents could accurately name and describe their child’s heart disease [18]. Attention-narrowing hypothesis states that individuals in stressful situations will often focus on the “threatening” or central information at the cost of remembering peripheral information [15–17]. This has been described in oncology populations with focus on diagnosis but inability to recall management and treatment goals [15–17]. Previous studies have shown that women presenting for fetal echocardiogram have significantly higher level of anxiety, which in turn may effect memory [17, 19, 20]. Thus, it is possible that parents in the current study experienced a similar phenomenon with good understanding of their child’s diagnosis and immediate postnatal care needs with less recall of the longer-term outcomes.

This finding also underscores important clinical implications. Fetal counseling guides highlight many topics for discussion; however, communication and understanding may be limited by the sheer volume of information coupled with parental stress during these initial discussions. As efforts continue to enhance patient- and family-centered fetal counseling, it will be important to include diverse voices and perspectives as to which topics are most important to cover during the first consultation, while also involving parents in follow-up consultation as they go through the pregnancy. Adult studies have found that physicians often fail to recognize patients’ misconceptions regarding their diagnosis, health literacy or preferences for information [10, 21–25]. This was not the case in our study. Parental subjective understanding and the cardiologists’ perception of parental understanding were consistent with excellent congruence for fetal diagnosis and fair congruence with long-term outcomes. While cardiologists and most parents perceived good parental understanding of topics regarding long-term outcomes such as risk of mortality, objective knowledge acquisition may not be adequate. These results call for cardiologists to reevaluate parental understanding of previously discussed topics, especially those associated with long-term outcomes regardless of parental perceived understanding.

Despite knowledge gaps, all parents were satisfied with their initial counseling session and felt that it met their needs. This is in contrast to a previous cross-sectional survey analysis of older children with complex CHD that found that parents reported that they would have preferred more information during fetal consultation than cardiologists provided [6, 9]. To our knowledge, this is only study to survey parents during the pregnancy about whether counseling met their needs. Our results suggest trying to cover an exhaustive list of topics may not be needed, nor appropriate, for every patient encounter at the time of diagnosis. Parental knowledge needs are likely to change throughout the heart disease course. This emphasizes a need for personalized assessment of communication preferences and needs to best inform counseling practices.

This study has several limitations. Being a single-center study, its findings may not be generalizable to the broader prenatal counseling population for congenital heart disease (CHD). Only 51% of eligible parents completed the surveys. Despite the low response rate, participants and non-participants were demographically similar, except for differences in maternal age, gestational age, and insurance status. Our participants primarily spoke English and were highly educated, with 50% being college graduates and 28% holding graduate degrees. Various other survey studies have found that patients with higher interest in participating in research were more likely to be younger, female, more educated, and/or have higher health literacy [21, 26]. Previous studies have found that parent education levels were associated with improved parental understanding of CHD [9, 10, 18, 25, 27]. These studies suggest that our findings may be an overestimate of parental understanding of topics commonly discussed in prenatal counseling sessions. Families less overwhelmed by new information are probable more likely to participate in the study. Our survey followed the first pediatric cardiology visit, though variation likely existed in the obstetrician’s consultation about CHD at referral. Parental and cardiologist recall bias is possible due to the time between the initial prenatal visit and the questionnaire. However, this bias is likely less significant than in studies analyzing prenatal counseling months or years after birth [4, 9, 18]. Parental acquisition of knowledge at counseling visits and understanding of CHD has not been extensively studied, thus a standardized assessment tool is not available.

Future directions of this project include reevaluation of parental understanding after additional prenatal counseling visits, as patients with major CHD often have two to three prenatal visits prior to delivery, and whether there objective and perceive understanding improves. Furthermore, it would be interesting to evaluate parental understanding and satisfaction regarding prenatal counseling prior to discharge from the hospital and what topics they wish were included. Additionally, we know that just a referral for a fetal cardiology evaluation results in increased parental anxiety [5]. It would be interesting to evaluate parental perception of counseling of normal findings on fetal echocardiogram such as premature atrial contraction or echogenic foci as parental perception of child wellness may affect future interaction raising their child.

## Conclusion

For many aspects of the first prenatal counseling visit, post-visit parental perception of understanding is congruent with cardiologists’ perceptions of parental understanding; however, gaps exist in objective parental knowledge of long-term outcomes such as mortality and neurodevelopment outcomes. Our results suggest providers should reevaluate parental understanding of previously discussed topics, especially those associated with long-term outcomes regardless of parental perceived understanding.

## Summary

### “What’s Known on This Subject”

- Years after diagnosis, parents of children with congenital heart disease report wanting more information during prenatal consultation than was provided by cardiologists. However, studies in other patient populations suggest much of the information provided may not be retained.

### “What This Study Adds”

- This study assessed both perception of understanding and knowledge acquisition in parents of fetuses with complex congenital heart disease shortly after initial consultation with a pediatric cardiologist.

## Supplementary information

Parental and Cardiologist surveys

## Data Availability

The datasets generated during and/or analyzed during the current study are not publicly available due persistent identifiers such as date of care and date of birth, but are available from the corresponding author on reasonable request.
