# Supplementary material for: Prenatal counseling for heart disease: Perception of understanding and communication gaps
Source: J Perinatol. 2025 Aug 21;45(9):1207–12. doi: 10.1038/s41372-025-02365-3 (PMC12431847; doi:10.1038/s41372-025-02365-3)
Supplement: Supplementary file 1 — Parental and Cardiologist surveys [file 41372_2025_2365_MOESM1_ESM.docx]

**Parental Questionnaire:**

Timing: parental questionnaire will be administered at the follow-up prenatal visit which occurs 4-6 weeks after the initial prenatal visit.

**Data Populated by Study Team:**

1. Diagnosis (based on initial cardiologist survey)
2. Gestational age at first UM visit
3. Maternal age
4. Marital status
5. Family history of congenital heart disease
6. Insurance (public vs private)

**Patient Questions:**

1. **First Question:** In your own words, tell us the name of your baby’s heart problem or give a description of it: _______________________

**Demographics and Information regarding first visit:**

1. Are you the mother of the baby? Yes/No
   1. If No: What is your relationship to the baby?
      1. Father
      2. Spouse/partner
      3. Grandparent
      4. Other:____________
   2. If No: How old are you? ________ years
2. What is your highest completed level of education?
   1. have not completed high school
   2. high school
   3. technical school (after high school)
   4. college
   5. graduate school
   6. other:___________

The following questions ask about the time before your first visit to the Michigan Medicine Prenatal Heart Program:

1. Before your first visit to the Michigan Medicine Prenatal Heart Program, did you meet with any doctors about your baby’s heart? Yes/no/I don’t know
   1. If yes: what kind of doctors had talked to you about your baby’s heart (may select more than one)?
      1. Obstetrician
      2. Maternal-fetal Medicine Specialist
      3. Pediatric Cardiologist
      4. Other: _________
      5. I don’t know
2. Before your first visit to the Michigan Medicine Prenatal Heart Program, did you look for additional sources of information about your baby’s heart (ex. websites, social media)? Yes/No/I don’t know
   1. If yes: what sources of information did you use (select all that apply)
      1. Medical websites
      2. Support group websites
      3. Social Media (Facebook, etc)
      4. Other: ____________

The following questions ask about your ***first*** visit to the Michigan Medicine Prenatal Heart Program:

1. Did anyone come with you to your first visit to the Michigan Medicine Prenatal Heart Program? Yes/No
   1. If Yes: Who came with you?
      1. Father of the baby
      2. Spouse/partner
      3. Friend
      4. Your parent
      5. Children. If so, how many and ages
      6. Other: ____________
2. At your first visit to the Michigan Medicine Prenatal Heart Program did you receive any written information about your baby’s heart (select all that apply)
   1. Diagram of my baby’s heart
   2. Care Notebook (*can we insert a picture?)*
   3. Family Counseling Checklist (*can we insert a picture?)*
   4. Other: __________

The following questions ask about your understanding of your baby’s heart problem:

1. I feel that I understand my baby’s heart problem after being counselled at my first visit.
   1. Strongly agree
   2. Agree
   3. Neither agree nor disagree
   4. Disagree
   5. Strongly disagree
2. Does your baby have any problems in addition to the heart problem (ex. chromosome problems, problems with other organs)? Yes/No/I don’t know
   1. If Yes: what problems does your baby have? ____________
3. Will your baby need heart surgery? Yes/No/I don’t know
   1. If Yes: how many heart surgeries will your baby most likely need during his/her lifetime?
      1. 1
      2. 2
      3. 3
      4. More than 3
      5. I don’t know
4. The following questions ask about your understanding of the treatment for your baby’s heart problem?
   1. Despite my baby’s heart problem, the fetal heart doctor stated that I could deliver my baby at my local hospital.
      1. Yes
      2. No
      3. I don’t know
      4. We did not discuss this at the previous visit
   2. My baby will have to be admitted to the intensive care unit shortly after being born.
      1. Yes
      2. No
      3. I don’t know
      4. We did not address this at our previous visit
   3. My baby will be able to go home from the hospital before they need heart surgery.
      1. Yes
      2. No
      3. I don’t know
      4. We did not discuss this at my previous visit
   4. My baby’s heart problem can be completely cured with heart surgery.
      1. Yes
      2. No
      3. I don’t know
      4. We did not discuss this at my previous visit
   5. My baby’s heart problem can NOT be cured with heart surgery, but can be treated by a series of surgeries.
      1. Yes
      2. No
      3. I don’t know
      4. We did not discuss this at my previous visit
5. The following questions ask about your understanding of how your baby’s heart problem will affect them life-long?
   1. My baby’s heart problem will require life-long cardiology care.
      1. Likely
      2. Unlikely
      3. I don’t know
      4. We did not discuss this at my previous visit
   2. Despite medical and surgical treatment, some children with my baby’s heart problem do not survive.
      1. Likely
      2. Unlikely
      3. I don’t know
      4. We did not discuss this at my previous visit
   3. Even with heart surgery, my child may not be able to be as active as other children.
      1. Likely
      2. Unlikely
      3. I don’t know
      4. We did not discuss this at my previous visit
   4. My child may have an increased risk of learning disabilities and developmental delays related to his/her heart problem and heart surgeries
      1. Likely
      2. Unlikely
      3. I don’t know
      4. We did not discuss this at my previous visit
6. The following questions ask about the amount of information who feel you have about your baby’s heart problem? *Answers for all subquestions are: Strongly agree, agree, neither agree nor disagree, disagree, strongly disagree. If disagree or strongly disagree, ask: I wish I had more information about_____________*
   1. I have gotten enough information about my baby’s heart problem
   2. I have gotten enough information about the care my baby will need after delivery
   3. I have gotten enough information about the long term outcomes of babies with my baby’s heart problem
7. Since your visit to the Michigan Medicine Prenatal Heart Program, did you use any of the following sources of information about your baby’s heart?
   1. Medical websites
   2. Support group websites
   3. Social Media (Facebook, etc)
   4. Other: ____________
8. How would you rate the quality of fetal counseling visit you have received.
   1. Excellent
   2. Good
   3. Fair
   4. Poor
9. Did you get the kind of service you wanted in your fetal counseling appointment?
   1. No
   2. definitely not
   3. not really
   4. Yes generally
   5. Yes definitely
10. To what extent has our program met your needs?
    1. Almost all of my needs have been met
    2. Most of my needs have been met
    3. Only a few of my needs have been met
    4. None of my needs have been met
11. How satisfied are you with the amount of help you have received?
    1. Quite dissatisfied
    2. Indifferent or mildly dissatisfied
    3. Mostly satisfied
    4. Very satisfied
12. In an overall, general sense, how satisfied are you with the fetal counseling visit?
    1. Very satisfied
    2. Mostly satisfied
    3. Indifferent or mild dissatisfied
    4. Quite dissatisfied
13. If you were to seek help again, would you come back to our program?
    1. No definitely not
    2. No, I don’t think so
    3. Yes, I think so
    4. Yes, definitely
14. *If dissatisfied, please explain if willing.*

**Provider Questionnaires**

Done on iPad during or immediately after the initial consultation

**APN Questionnaire:**

1. Fetal Cardiac Diagnosis:
2. Cardiologist initials
3. Other non-cardiac diagnoses: Yes: ___________ No
4. Gestational Age: __________ wks _______ days
5. Who was at the counselling session (select all that apply)
   1. Mother
   2. Father of baby
   3. Fetal grandparent
   4. Friend
   5. Children
      1. Approximate age
   6. Other: ______________
6. The mother has additional appointments the same day with the following people (select all that apply)
   1. FDC
   2. High Risk OB clinic
   3. Cardiac Surgery
   4. Social work
   5. Child life (had a tour)
   6. Other: __________
   7. No other appointments
7. Has the mother met with another pediatric cardiologist prior to this appointment? Yes/No/IDK
8. Time counselling started: *(approximately how many minutes)*
9. Time Counselling ended: *(approximately how many minutes)*
10. How much time did you spend with family after or before they met with the cardiologist? *(approximately how many minutes)*
11. Parents were provided the following written resources at their appointment.
    1. Normal cardiac diagram
    2. Congenital heart disease diagram
    3. Care notebook
    4. Mended Little Hearts Bag given
    5. Family Counseling checklist
    6. Other:_______
12. Please check if the following topics were discussed at the prenatal appointment:
    1. Fetal Cardiac Diagnosis
    2. Diagnosis will require Mom’s to deliver infant at UM
    3. PCTU admission after delivery
    4. Diagnosis will require neonatal surgical repair
    5. Number of surgeries life-long
       1. 1
       2. 2
       3. 3
       4. More than 3
    6. That cardiac surgery is palliative and not a curative procedure.
    7. Due to cardiac diagnosis, their infant will require life-long cardiac care.
    8. Despite medical and surgical care, some children with that specific cardiac diagnosis do not survive.
    9. That the infant’s diagnosis may result in decreased exercise tolerance.
    10. Increase risk for neurodevelopmental delays associated with congenital heart disease.
13. What is your sense of the mother’s understanding of the following things: *Strongly agree, agree, neither agree nor disagree, disagree, strongly disagree. If disagree or strongly disagree: what barriers to understanding did you identify (ex. mother not feeling well, counselling time cut short, etc): ____________*
    1. Mother had a good understanding of fetal diagnosis after this visit
    2. Mother had a good understanding of initial hospitalization after this visit
    3. Mother had a good understanding of long term outcomes of this fetal diagnosis after this visit.
    4. Mother was satisfied with the consultation
14. Did you think the other family members/friends at the consultation have a similar understanding as the mother? *Yes/No/I don’t know/Free text box*.
    1. If No: Do you think they understood: A lot more, more,, a lot less, I don’t know, free text box
15. Any other obstacles of parental understanding that you faced during counselling?
    1. Time constraints
    2. Decreased health literacy
    3. Family focus on a specific topic
       1. What topic
    4. Parental anxiety
    5. External factors (ie. Young children, lots of family in session)
    6. Had to leave to get to another appointment
    7. Parents had other questions that needed to addressed
       1. Topic of questions
    8. Other: (fill in the blank)

**Cardiologist Questionnaire**

1. Please write your initials
2. Fetal cardiac diagnosis:
3. Please check if the following topics were discussed at the prenatal appointment:
   1. Fetal Cardiac Diagnosis
   2. Diagnosis will require Mom’s to deliver infant at UM
   3. PCTU admission after delivery
   4. Diagnosis will require neonatal surgical repair
   5. Number of surgeries life-long
      1. 1
      2. 2
      3. 3
      4. More than 3
   6. That cardiac surgery is palliative and not a curative procedure.
   7. Due to cardiac diagnosis, their infant will require life-long cardiac care.
   8. Despite medical and surgical care, some children with that specific cardiac diagnosis do not survive.
   9. That the infant’s diagnosis may result in decreased exercise tolerance.
   10. Increase risk for neurodevelopmental delays associated with congenital heart disease.
4. Choose the number of surgeries that you anticipate this baby will need over his/her lifetime (ex. 3 surgeries for single ventricle palliation):
   1. 1
   2. 2
   3. 3
   4. More than 3
5. Give an estimated survival to initial hospital discharge for this baby assuming a term delivery. Please choose one option (use comments for circumstances unique to this baby that alter your estimate):
   1. < 50%
   2. 50-80%
   3. 80-90%
   4. 90-95%
   5. > 95%
   6. Comments: _____________
6. Give an estimated survival to 5 years of age for this baby assuming a term delivery. Please choose one option (use comments for circumstances unique to this baby that alter your estimate):
   1. < 25%
   2. 25-50%
   3. 50-75%
   4. 75-95%
   5. > 95%
7. What is your sense of the mother’s understanding of the following things: *Strongly agree, agree, neither agree nor disagree, disagree, strongly disagree. If disagree or strongly disagree: what barriers to understanding did you identify (ex. mother not feeling well, counselling time cut short, etc): ____________*
   1. Mother had a good understanding of fetal diagnosis after this visit
   2. Mother had a good understanding of initial hospitalization after this visit
   3. Mother had a good understanding of long term outcomes of this fetal diagnosis after this visit.
   4. Mother was satisfied with the consultation
8. Did you think the other family members/friends at the consultation have a similar understanding as the mother? *Yes/No/I don’t know/Free text box*.
   1. If No: Do you think they understood: *A lot more, more, less, a lot less, I don’t know, free text box*
